# Towards Mapping of Soil Crust Using Multispectral Imaging

**DOI:** 10.3390/s21051850

**Published:** 2021-03-06

**Authors:** Giacomo Crucil, Kristof Van Oost

**Affiliations:** 1 TECLIM, George Lemaître Center for Earth and Climate Research, Earth and Life Institute, Université catholique de Louvain, 1348 Louvain-La-Neuve, Belgium; 2 Fonds de la Recherche Scientifique—FNRS Rue d’Egmont 5, 1000 Bruxelles, Belgium

**Keywords:** soil crusting, multispectral imaging, soil roughness, photogrammetry, rainfall kinetic energy

## Abstract

Soil crusts and surface roughness are properties which are highly dynamic in both space and time that change in response to biotic processes, meteorological conditions and farming operations. These factors, however, are difficult to quantify and are usually described using simplified expert-based classes. This hampers a clear identification of the controlling factors and their relation to soil erosion and sediment generation processes. The availability of new small portable multispectral cameras offers the potential to study soil surface dynamics at a high spatial and temporal resolution. The objective of this study was to analyse the relationship between soil crusting, represented by cumulative rainfall kinetic energy, and soil surface reflectance, as derived from vis-NIR multispectral imaging. We designed a series of rainfall-soil surface experiments to disentangle the effects of soil crusting on spectral reflectance factors from those related to surface micro-scale roughness. Partial least squared regression (PLSR) models were developed to predict both kinetic energy and roughness from multispectral images. We evaluated different roughness removal methods which were based on the transformation of reflectance through standard normal variate (SNV) and roughness thresholding using high resolution digital elevation models. Furthermore, we assigned the light scattering effect related to roughness in the multispectral spatial domain by calculating the inter-quantile range of the reflectance values in a kernel. Our experiments and workflow demonstrate that it is possible to model crust development, using rainfall kinetic energy as a proxy, from vis-NIR based multispectral imaging.

## 1. Introduction

There have been persistent issues in relation to the reliability of runoff and soil loss assessments and their scientific interpretations. A long-standing challenge in the field has been the observation that the cause of soil surface runoff is non-unique; i.e., the same apparent soil surface and rainfall conditions do not necessarily result in the same runoff and erosion rate [1,2,3]. This is convincingly demonstrated by data from replicate plots where the variability of runoff and erosion response reaches up to two orders of magnitude difference [4,5]. This feature makes the robust assessment of soil runoff/erosion and subsequently landscape management difficult; more importantly, this calls for further scrutiny of other contributing factors. Small-scale experimental [1,6,7,8,9] and modelling [10] have suggested that micro-scale properties of the first few centimetres of the soil surface strongly affects its infiltration capacity and erodibility. These studies showed that soil physical degradation from the changes in roughness and the development of soil crusts are important factors controlling the above-mentioned variability, particularly on fine-textured soils.

The term soil crusting refers to the forming processes and the consequences of a thin layer at the soil surface with reduced porosity and high penetration resistance, favouring runoff initiation and inter-rill soil erosion. Soil crusting can be monitored directly through morphological changes (e.g., the diameter of the smallest clods not incorporated in the crust, D_lim_), or indirectly through sealing indexes based on the decrease in infiltration capacity or increase in surface strength. In different studies, both D_lim_ and sealing indexes have been linearly related to cumulative rainfall kinetic energy [11]. Crusts are commonly classified in two subsequent stages, “structural” and “depositional”, respectively based on the origin of soil seal formation, as the result of two complementary mechanisms [12]: (i) physical disintegration of surface aggregates caused by wetting of the dry aggregates and/or the beating action of the raindrops, and subsequent compaction of the disintegrated aggregates by raindrop impact; (ii) the physicochemical dispersion of soil clays which migrate downward with the infiltrating water clogging pores. 

Contradictory evidence has been reported for the relationship between soil surface roughness and crust generation and for the effect of roughness on the generation of runoff [8]. The problem may reside in the fact that these two properties are highly dynamic in both space and time in response to biotic processes, the energetic input of rainfall and/or farming operations. Even under laboratory conditions, or controlled field experiments, these factors are difficult to quantify and are usually described using simplified expert-based classes [13]. Arguably, one of the reasons that erosion modelling is still challenging [14] is the lack of good input data that captures the heterogeneity of the soil surface characteristics. Therefore, one way to improve not only our mechanistic understanding of rainfall-runoff processes, but also erosion model performance, is to improve the accuracy and precision of model input data, using new sensor measurement methods, and to test how sensitive the model is to variations in input data [15]. 

In this context, soil spectral analysis was proven to be very effective in the estimation of a wide range of soil properties [16]. The spectral properties of crusted soils were already the subject of several studies looking at specific spectral features [17], and this was also related to soil infiltration rates and cumulative rainfall kinetic energy [18,19,20]. The recent advent of small portable sensors with multi-spectral capabilities now provide the means, the accuracy and the resolution to quantitatively and accurately assess spatial-temporal variability of soil conditions at different scales, especially if combined with low altitude flights with unmanned aerial systems (UAS) [21]. These systems have the potential to present a better alternative to ground surveys, on one side, and satellite data on the other: they offer superior spatial and temporal resolution at a better time- resource- and cost- efficiency. For example, Croft et al. in several studies [22,23,24] successfully developed and refined models of soil surface roughness using proximal hemispherical measurements of hyperspectral directional reflectance factors. Nonetheless, they recognized the difficulty of separating the contribution of soil variable like roughness, moisture, and organic carbon to soil reflectance factors, and the complexity that exists in attempting to retrieve them from optical remotely sensed data [25]. Elucidating the factors, as well their interactions, that shape the soil spectral signature is therefore critical. This is important in the context of developing predictive models of soil crust development suitable for proximal sensing applications and/or low altitude UAS flights.

The general objective of this study is to analyse the functional relationships between soil crusting and soil surface reflectance as derived from multispectral imaging and how this can be inferred from low-altitude UAS sensors. More specifically, we aim at disentangling the effects of soil surface roughness from those of crust development on spectral reflectance factors, by establishing and evaluating a dedicated data pre-processing protocol. To this end, we carried out indoor and outdoor experiments to monitor the evolution of soil crust and micro-scale soil roughness in response to cumulative rainfall kinetic energy. We combine data from a hyperspectral sensor for spectral feature characterization at high resolution with image analysis in the spatial domain using a vis-NIR multispectral camera and high-resolution photogrammetry. The dynamics of soil crusting and soil roughness development were studied at two spatial scales: (i) at the 2 mm scale, which is the size of the small soil clods affecting micro-scale roughness, and (ii) at the 15 mm scale, which is the resolution obtainable by low-altitude UAS flights.

## 2. Materials and Methods

### 2.1. Experiment Design and Data Series

**Figure 1:**
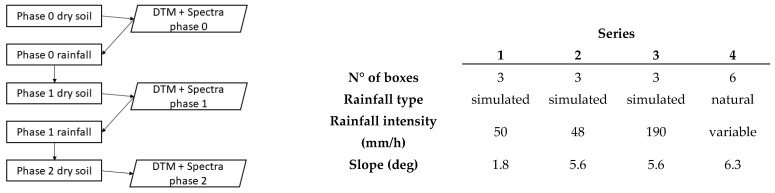
Data series structure (**left**) and variables (**right**).

Soil samples were subjected to two separate phases of simulated or natural rainfalls: the aim was to force the evolution of soil crust and changes in surface micro-scale random roughness while controlling rainfall input. The runoff coefficient (RC) was calculated for every rainfall event as the ratio between the amount of runoff and the amount of precipitation. Soil crust development was first quantified from the changes in runoff caused by the progressive surface sealing. The amount of rainfall kinetic energy (KE) previously applied to a soil sample to generate each specific crust condition was then used as a proxy for crust development. Surface conditions were sensed under dry soil conditions at the start of the experiment and after each rainfall application phase. Sensing was executed by means of close-up photogrammetry, multispectral imaging, and hyperspectral point measurements. This data was acquired to (i) estimate surface random roughness (RG) from a high-resolution digital terrain model (DTM) and (ii) to provide a spectral descriptor for crust development. In this way, both soil crusting and roughness were related as a response to cumulative rainfall kinetic energy. Four data series, consisting of three phases of surface monitoring, were generated to obtain a range of KE/RC/RG values. Three of the data series, each with 3 replicates, were obtained under laboratory conditions using simulated rainfall, while one data series, with six replicates, was obtained outdoor under natural rainfall. The data series structure is summarized in Figure 1.

### 2.2. Samples Setup

**Figure 2:**
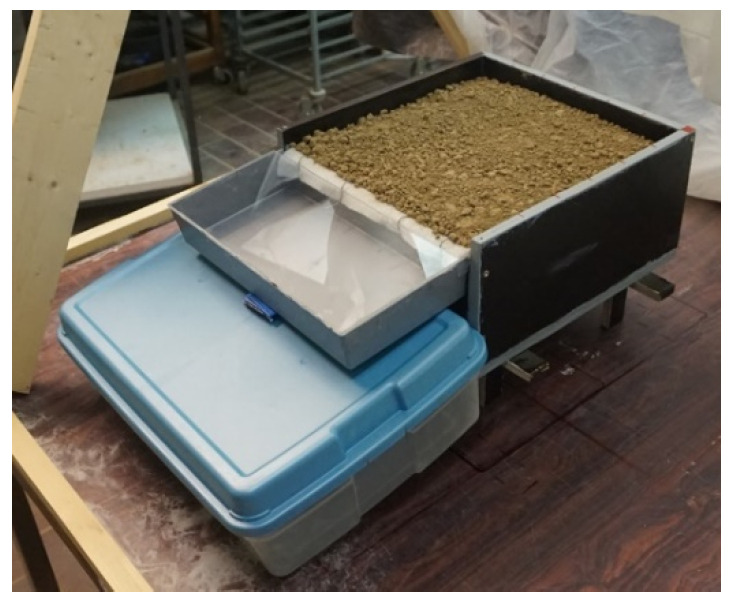
Illustration of a soilbox used during the experiments.

Soil samples were collected from two cultivated fields situated in the Belgian loam belt. They are classified as silt-loam by texture according to the USDA classification. Each container, from now referred to as “soilbox” (Figure 2), consists of a wooden box of 30 cm × 30 cm wide, 15 cm tall, open on the top and filled with 14 cm of manually compacted soil. The downslope side of the soilbox accommodates two sections that allow for water outflow: (i) on the top, a rectangular broad-crested weir, 30 cm wide and 2 cm tall, levelled with the soil for surface runoff: (ii) on the bottom, a rectangular hole 30 cm wide and 2.5 cm tall, for water exfiltration. A 2.5 cm thick metallic grate covered with geo-tissue was placed on the floor of the soilbox, acting like a selective membrane toward the exfiltration hole, to prevent soil loss while allowing water outflow.

### 2.3. Rainfall Data

An indoor rainfall simulator, consisting of an axial-flow full cone nozzle (Series 490/491 Lechler spray nozzles and engineered systems, Lechler GmbH, Metzingen, Germany) was positioned at a height of 3 m. The nozzle was connected to the water grid through a discharge gauge in order to set and monitor the amount of litres/second of water applied. A panel of 30 cm × 30 cm with 16 small buckets, was used to calibrate the rainfall simulator by converting the measured discharge to mm/h at floor level (after a drop fall of 3 m). Natural rainfall was measured with a tipping bucket rain gauge with a time resolution of 5 min and depth resolution of 0.25 mm. Rainfall intensity was kept constant during the simulated events and was variable under natural rainfall.

To estimate the combined effect of rainfall duration and intensity on the soil surface (i.e., the destruction of clods and particle displacement), cumulative kinetic energy was chosen as an indicator. To estimate the kinetic energy KE_p_ (J·m^−2^) applied during each measurement phase, time-specific rainfall kinetic energy KE_t_ (J·m^−2^·h^−1^) was first estimated using the universal power law proposed by [26], for each 5 min measuring time-step. KE_p_ was then estimated as the sum of KE_t_ for each measurement time step during a phase. This method is a simplification based on the assumption that the drop-size distribution is uniform in constant rainfall intensity. The cumulated kinetic energy KE was then estimated for each dry soil phase as the sum of each antecedent KE_p_ since the start of the experiment. 

### 2.4. Sensors

A Nikon D3100 camera (Nikon Inc., Tokyo, Japan) was used for photogrammetric soil surface reconstruction. The camera model is based on a DX format RGB CMOS sensor with a max resolution of 4608 × 3072 (14.2 effective megapixels). The camera was mounted with an 18-55 mm objective which was fixed at a focal length of 30 mm. The multispectral sensors employed was a Micasense Rededge-M multispectral camera (MicaSense Inc., Seattle, WA, USA). This camera is equipped with five imaging sensors filtering for spectral bands centred at 475, 560, 668, 717 and 840 nm wavelengths, with a bandwidth (FWHM) of 20, 20, 10, 40 and 10 nm respectively. The image resolution is 1280 × 960 pixels and the camera has a focal length of 5.4 mm. The hyperspectral sensor was an ASD Fieldspec 3 FR spectro-radiometer (Analytical Spectral Devices Inc., Boulder, CO, USA) that provides single-spot measurements of light intensity data in the vis-NIR-SWIR region (350–2500 nm) with an optical resolution of 3 nm in the 350–1000 nm region and 10 nm for the 1000–2500 nm region, all resampled at a 1 nm data output resolution. 

### 2.5. Roughness Characterization Using Photogrammetry

**Figure 3:**
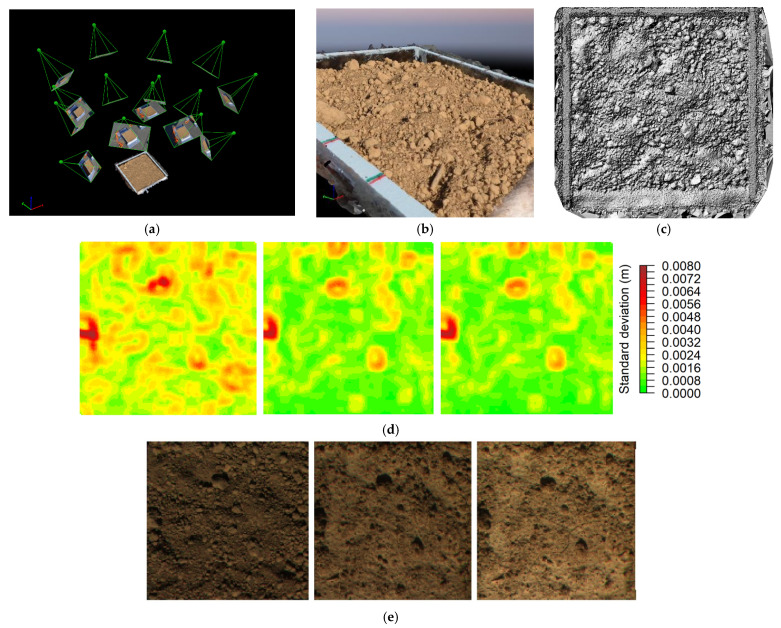
Roughness characterization using photogrammetry: (**a**) an illustration of the 3D mesh and the estimated positions of the camera; (**b**) detailed view of the 3D mesh from close distance; (**c**) DTM with hillshade representation. In the middle (**d**) an example of random roughness map, for the same soilbox, in three subsequent phases (left to right: before, after first and after second rainfall application) and, on the bottom (**e**), their corresponding RGB images.

For the photogrammetric surface reconstruction, 16 images were taken with the Nikon camera from different positions regularly distributed in a dome above the soil box, between 0.5 and 1 m of distance (see Figure 3a). A DTM with 0.2 mm resolution was then generated for each soilbox/phase with the software Pix4D [27] (Figure 3c), then resampled to 2 mm for calculation purposes. Four ground control points marked on the wooden frame of each soilbox were used to increase the accuracy of the DTMs and to allow the subsequent alignment of the multispectral images with the custom coordinate system of the surface elevation models. Geolocation accuracy of the control points was estimated at 1, 1, and 0.5 mm for the X, Y and Z components, respectively. The wooden frames were then cropped out of the DTMs. For each DTM, the residual topography (the DTM detrended from the effect of the general soilbox inclination) was extracted subtracting the plane of the average soilbox slope from all DTM elevations. Maps of random roughness (RG) were then generated by calculating the standard deviation of the elevations on the residual topography in a moving square window of 21 mm × 21 mm (example of roughness map series in Figure 3d, and corresponding RGB images in Figure 3e). The average value of random roughness for the whole box window was calculated in the same way.

### 2.6. Spectral Data Acquisition and Pre-Processing

Soil spectral measurements were carried out in outdoor conditions in an open area to minimize reflection from vertical objects in the surroundings, during stable, clear-sky and natural sunlight conditions for the whole duration of the measurements. Measurements were taken within two hours of solar noon. Before every sensor measurement took place, the soil-boxes were oven-dried at 60 °C for 72 h and then air-dried in open air for at least other 48 h. For the hyperspectral measures, the sensor head was set at c. 6 cm height above the soil surface, at the nadir position, providing a measurement footprint with a diameter of c. 2.6 cm. This footprint was chosen to allow for an easy individuation on the multispectral images for spectral comparison. Each single spectral acquisition with the ASD instruments was performed with an integration time of 5.4 s. 25 measurements were taken during each phase, in a regular 5 cm × 5 cm grid. Radiometric calibration for the ASD was automatically managed by its hardware + software routine. For the conversion of radiance to reflectance a Spectralon white reference surface measurement was repeated every ten samples. Bandwidths below 400 nm and above 2400 nm were removed because they typically contain excessive noise, as well as the bandwidths influenced by water vapour absorption in the intervals 1350–1450 and 1800–1950 nm. Spectra were also smoothed with a Savitzky-Golay smoothing filter [28] using a second order polynomial fit. Spectral outliers were removed, at each box/phase level, setting a threshold of three times the standard deviation of the Mahalanobis distance of the spectra projected in the first two latent variables of the principal component space. The average of the 25 spectra (minus outliers) was then used as representative for each box/phase. 

For multispectral imaging, a single picture was taken for each soilbox phase, from 1 m distance at the nadir position. Radiometric calibration, image correction and conversion to reflectance for the Rededge-M images were performed in post processing following the procedure described on the manufacturer’s website [29] using the software R [30]. Conversion to reflectance was possible thanks to the employment of a calibrated reflectance panel (provided by the Rededge-M manufacturer MicaSense Inc., Seattle, WA, USA), which was sampled before each sample image acquisition. Reflectance images were then scaled to true dimension (resulting in pixel size of c. 0.5 mm) and orthorectified by anchoring the 4 ground control points visible in the pictures to those identified in the photogrammetric procedure, using R software and gdal [31] scripts. Scaling and orthorectification were executed separately for each image in the 5 spectral bands to account for the different offset of the Rededge-M sensors. Finally, images were resampled at two resolutions: 2 mm and 15 mm.

**Figure 4:**
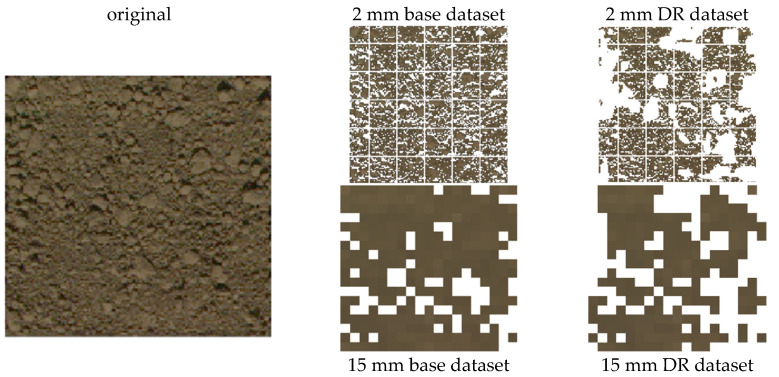
Example of RGB composite from multispectral image that shows the steps of pixel selection for the selective extraction of spectral data: original image; base dataset (with shadows removed) at 2 mm and 15 mm resampling; DR dataset (with roughness threshold < 2 mm) at 2 mm and 15 mm resampling. The images at 2 mm resolution also display with white lines the 6 × 6 grid used for partitioning reflectance histogram extraction.

Since multispectral images were acquired at very high resolution, shadows cast on the soil surface were affecting a substantial portion of the soil surface. Shadows commonly cause partial or total loss of radiometric signature, hence shadow reduction or removal becomes important during image analysis [32]. A threshold segmentation method applied on the intensity (also referred as brightness) component I of the HSI colour system [33] was calibrated and found to be sufficient for shadow detection and elimination on our case study. The method is discussed in detail in the Appendix A. Shadows from micro-reliefs and soil cracks were removed from all multispectral images with this method to create the base dataset (example in Figure 4, central column). Furthermore, a new set of multispectral maps (“DR”) was generated by removing pixels where the random roughness was above the threshold of 2 mm, which was visually assessed as appropriate to discard soil clods, and consistent with the minimum pixel size (example in Figure 4, right columns). The underlying idea is that this allows isolating soil crust colour information from the light scattering effect of roughness.

### 2.7. Multispectral Data Extraction

**Figure 5:**
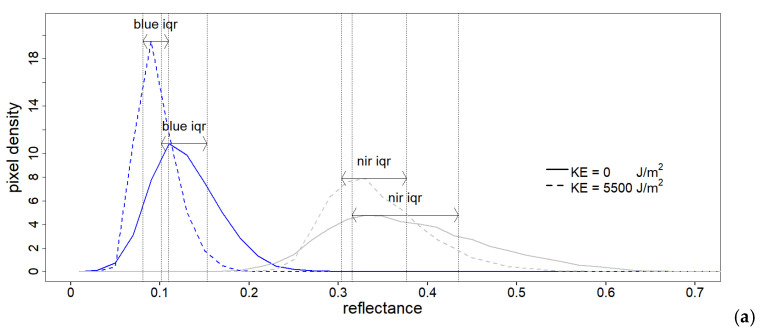

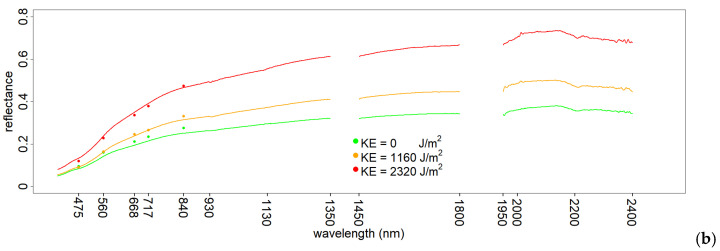
(**a**) Example of the evolution of the multispectral pixel density histograms, for two spectral bands drawn as an example (blue and nir, respectively colored in blue and grey), from a single soilbox across two different amounts of cumulated rainfall kinetic energy (0 and 5500 J/m^2^). The iqr is visualized as the span distance between the 1st and 3rd quantile of each histogram; (**b**) example of the evolution of mean multispectral reflectance values (points) and corresponding hyperspecral reflectance samples (lines) at the same soilbox location, for three levels of KE (0, 1160 and 2320 J/m^2^).

Previous studies [23,24] on the directional reflectance properties of soil surface showed that rough soils exhibit higher degrees of spectral anisotropy, resulting in wavelength-dependent spectral variability. We thus hypothesise that the residual (after de-shadowing) light scattering effect related to soil surface irregularities (i.e., roughness) in the multispectral spatial domain could be assigned to a spectral variability index in its spatial domain. We hereby propose to calculate this variability with a wavelength-specific inter-quantile range (iqr, visualized in Figure 5) of the reflectance values on a given surface. To do so, local spectral histograms for all the multispectral bands were extracted at two spatial scales, on each soilbox,: (i) in a 5 cm × 5 cm grid (example in Figure 4, top-right) on the images at 2 mm resolution (total of 36 squares per soilbox, number of total sampling locations n = 36 × 42 = 1512); (ii) on the whole soilbox for the images at 15 mm resolution (number of sampling locations n = 42). From each histogram two indexes for each of the five available broad-bands were used to summarize the multispectral data: the mean reflectance value and the inter-quantile range (iqr). This information was extracted both from the base dataset and from the DR one. Mean multispectral values were also compared for consistency with hyperspectral values at the same sampling locations (Figure 5b).

### 2.8. Spectral Modelling

The aim of the spectral data acquisition was to build a spectral model for soil crust development and to disentangle the effects of random roughness from soil physical-chemical changes on reflectance factors. In this framework, crust development (through its proxy kinetic energy) and roughness were used as the main target variables for partial least squared regression (PLSR) spectral modelling. Hyperspectral-based models were only used to assess the relative relevance of VIS, NIR and SWIR spectral bands in KE models through the variable importance in projection (VIP) score analysis and to motivate the choice for VIS-based multispectral data. Multispectral-based models were developed and validated to find the optimal data pre-treatment techniques for KE and RG prediction and mapping purposes.

From the hyperspectral data, PLSR models were developed on both raw reflectance (refl) and reflectance transformed through standard normal variate (SNV), using the R package “pls”. Standard normal variate normalizes each vector of spectral data by subtracting its mean and dividing by its standard deviation. It is intended to normalize spectral data to correct for light scattering, normally influenced by irregular surface geometry and particle size. In this study, its intended purpose was to understand to what degree it could be used to separate colour features from roughness features. 

From the multispectral data, PLSR models were developed on datasets that were filtered and/or transformed to remove the effect of roughness on spectral features in a gradual way: (i) base reflectance maps; (ii) reflectance maps with DR filter; (iii) SNV transform; (iv) SNV transform and DR filter. Models were developed at a 2 mm and 15 mm resolution. A “leave-one-out” cross validation was used to select the optimal number of latent variables for each sub-dataset. The best PLSR model was selected based on the number of latent variables (PLSR components) above which a decrease in RMSE was not significative. To evaluate the stability and consistency of the models a validation was then performed splitting each dataset into calibration and validation (80% and 20%, respectively), with random selection and model re-calibration for 200 repetitions. The performance of the models was evaluated on the validation datasets using the mean of 200 simulations of the following metrics: (i) relative error percentage (RE%), (ii) root mean square error of prediction (RMSE), (iii) coefficient of determination (R^2^) and (iv) ratio of performance to interquartile range (RPIQ). The VIP was used to assess variable importance in the PLS regressions. A threshold VIP score of 1 was used to highlight relevant variables (ref). 

**Figure 6:**
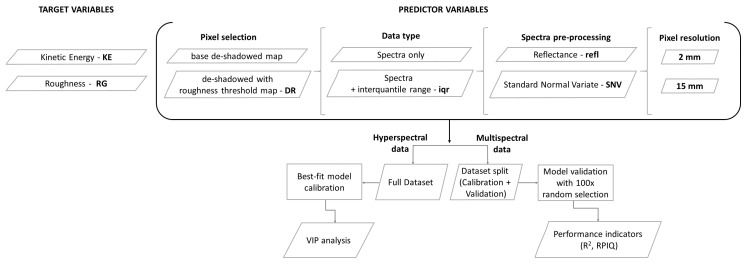
Flowchart of data selection and sub-setting for the PLSR modelling of multispectral data.

The modelled datasets are summarized in flow chart (Figure 6). 

## 3. Results and Discussion

### 3.1. KE, Crusting and Roughness Data

**Figure 7:**
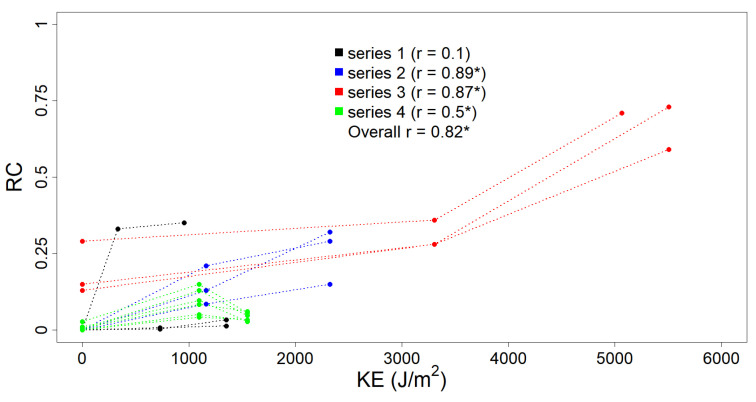
Scatterplot of KE in J/m^2^ and runoff coefficient RC. The pertinence to the four data series is highlighted with colours. Correlation values with *p* < 0.05 are marked with *.

Figure 7 shows the relationship between the runoff coefficient (RC) and the amount of kinetic energy KE previously received by the soil samples surface during the experiments. RC was positively correlated with KE, with an overall significant correlation of 0.82. These results are in line with the evidence, provided by previous studies [11,17], that the amount of KE received by a soil surface can be linked to the sealing effects and decrease in infiltration rate due to the development of a soil crust. This provides, together with the observed changes in soil spectra discussed below, a solid basis to link soil crust development to cumulative rainfall kinetic energy. 

**Figure 8:**
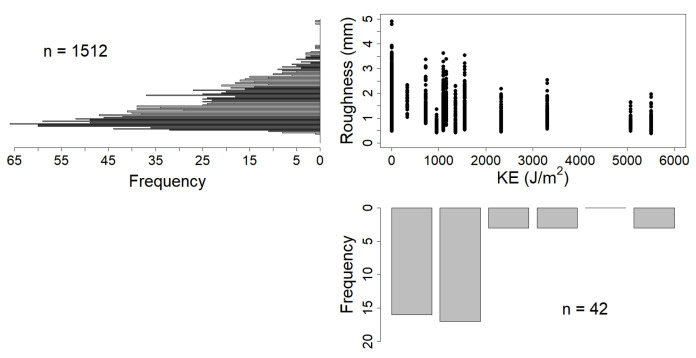
Scatterplot of KE in J/m^2^ and measured roughness in mm of standard deviation, detailed for the 2 mm resolution. Data histograms are plotted side by side to the respective axes.

**Figure 9:**
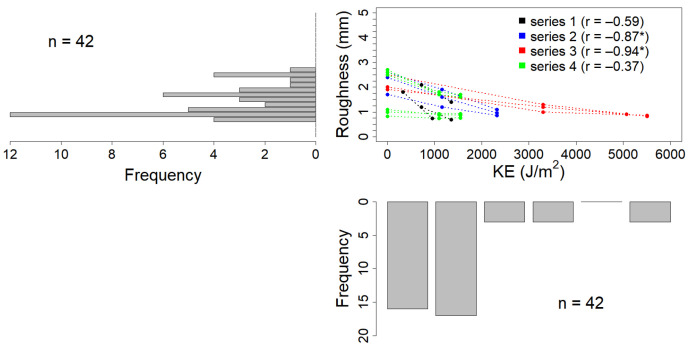
Scatterplot of KE in J/m^2^ and measured roughness in mm of standard deviation, detailed for the 15 mm resolution. Data histograms are plotted side by side to the respective axes. The pertinence to the four data series is highlighted with colours. Correlation values with *p* < 0.05 are marked with *.

Figure 8 and Figure 9 synthesizes the data obtained for KE and roughness for the two resolutions (i.e., 2 and 15 mm, respectively), and their relationship during the experiments. Both scatterplots show that the soilboxes were prepared with a range of starting roughness conditions (i.e., between 0–5 mm and 0 J/m^2^ of KE) that were subsequently levelled by rainfall to values between 1 and 2 mm. This resulted in a roughness distribution skewed toward these values, but with significant correlations (0.37 < r < 0.87) for all four data series. One data series (3rd) provided data with KE amount higher than 3000 J/m^2^, resulting in skewed distribution also for KE values. 

### 3.2. Hyperspectral Variable Importance

**Figure 10:**
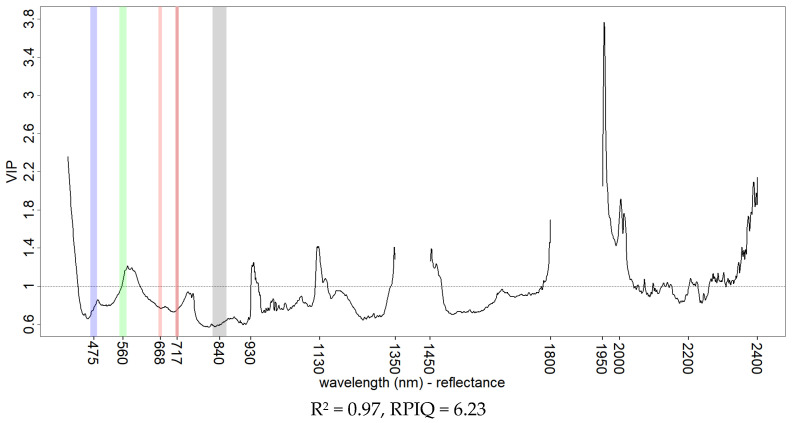
VIP scores of KE PLSR models from hyperspectral data using full vis-NIR-SWIR reflectance spectral range. Goodness of fit is reported using R^2^ and RPIQ. The multispectral broad-bands of the Rededge-M camera model are shown for comparison.

**Figure 11:**
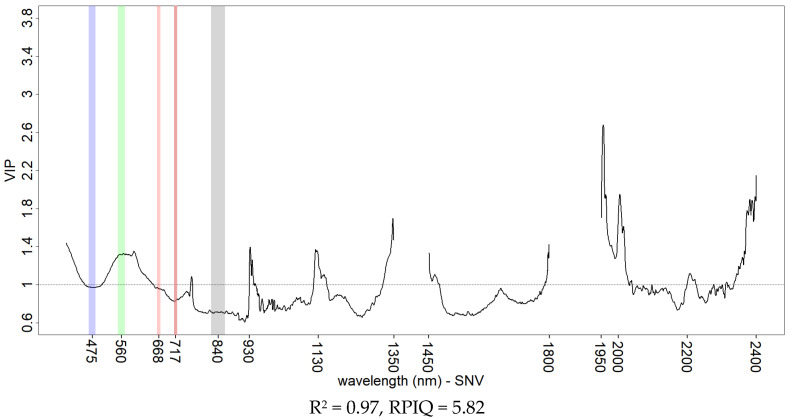
VIP scores of KE PLSR models from hyperspectral data using the SNV of the full vis-NIR-SWIR spectral range. Goodness of fit is reported with R^2^ and RPIQ. The multispectral broad-bands of the Rededge-M camera model are shown for comparison.

**Figure 12:**
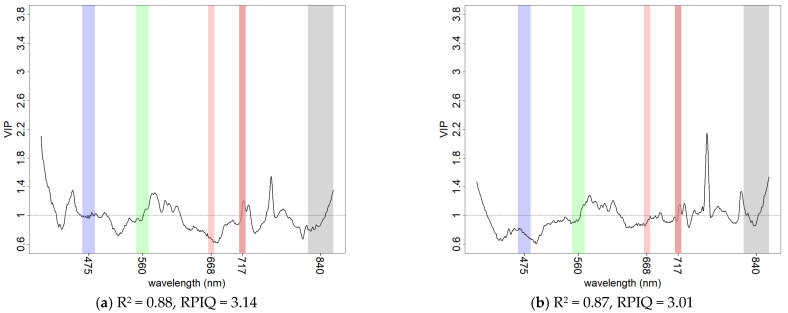
VIP scores of KE PLSR models from hyperspectral data using: (**a**) reflectance vis-NIR range; (**b**) SNV vis-NIR range. Goodness of fit is reported with R^2^ and RPIQ. The multispectral broad-bands of the Rededge-M camera model are shown for comparison.

PLSR models based on the hyperspectral data were developed aiming at the best fit to highlight the optimal variable importance for KE with different datasets. Full range reflectance data (Figure 10) provided the best model fit but the full range SNV (Figure 11) attributed more importance to the vis-NIR portion of the spectrum with comparable performance. Common peaks of information were found at 570, 930 and 1130 nm for the vis-NIR region and 1950, 2000, 2140 and 2200 nm for the SWIR region. The 2140 and 2200 nm peaks were already individuated and described by other studies [17,18] working on the spectral properties of soil crust and assigned to the enrichment of soil surface in clay particles during the crusting process. The use of only the vis-NIR portion of reflectance spectra (Figure 12a) and SNV (Figure 12b) with hyperspectral data showed that modelling of KE was possible, thus motivating the prospect of using the vis-NIR range only, in both base reflectance and SNV transformation. No sensible reflectance/SNV difference was obtained in either variable selection (except a peak on the blue reflectance wavelengths) nor performance, and a peak on information was observed in both datasets at 762 nm. An increase in baseline (albedo) was generally observed with increasing levels of KE (as reported in the example in Figure 5), in line with the observations made by the studies just mentioned [17,18]. Nevertheless, changes in slope in the vis-NIR portion of our soil spectral may have played an important role in KE model performance. These same studies [17,18] also pointed out that reflectance changes in soil crust are dependent on soil texture and mineralogy; the soil samples used in this study were homogeneous in texture and collected from the same area. Although this allows us to compare the different experiments, it represents a limiting factor for the generalization of our results.

### 3.3. Correlation with Multispectral Data

**Figure 13:**
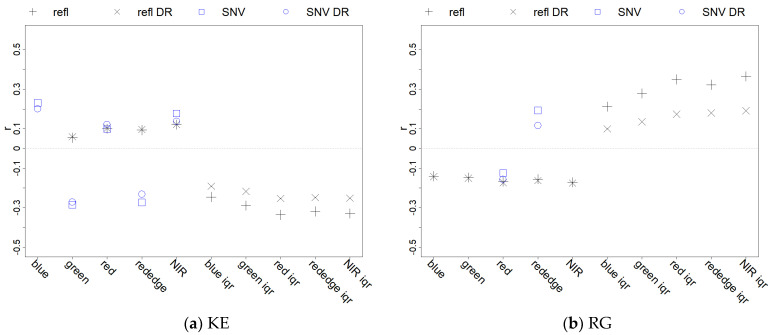
Correlation of the multispectral indexes with KE (**a**) and with roughness (**b**) from the 2 mm resolution dataset: “refl” represents the base dataset, “DR” the roughness threshold dataset, “SNV” the Standard Normal Variate transformation of reflectance data. Points where *p* < 0.05 were excluded from the plot.

A correlation analysis was carried out between the target parameters (i.e., KE and roughness) and the spectral indices (i.e., spectra and iqr) with different data pre-treatments (base, roughness thresholding DR, SNV) at the 2 mm spatial scale (Figure 13a,b, for KE and roughness, respectively). Considering only the results where *p* < 0.05, four base reflectance values (excluding the blue band) showed a low positive correlation with KE (0.03 < r < 0.12) and a low negative correlation with roughness (−0.13 < r < −0.17). On the other hand, iqr values showed significantly higher correlations, both negatively with KE (−0.24 < r < −0.33) and positively with roughness (0.21 < r < 0.36). In general, base reflectance and iqr correlation resulted in opposing trends between KE and roughness. The SNV transformation of base reflectance increased the correlation of some spectral bands with KE (blue = 0.23, green = −0.28, rededge = −0.27). SNV mildly affected the significative correlation of two spectral bands with roughness (red = −0.12, rededge = 0.19, with opposite sign respect to KE) and showed no correlation with the other 3 bands. The DR pre-treatment, with both KE and roughness, did not influence the correlation values of base reflectance or SNV but had a sensible effect in lowering the correlation of iqr (−22/24% with KE, −44/50% with roughness). This analysis shows that KE and roughness are spectrally entangled. This is also evident from the high correlation of their change after rainfall application. Nevertheless, the analysis shows that the iqr index seems to be a better index to describe roughness, due to its overall significant positive correlation and to its negative response to DR pre-treatment. SNV, on the other hand, was more strongly related to KE.

### 3.4. Modelling Multispectral Data

**Figure 14:**
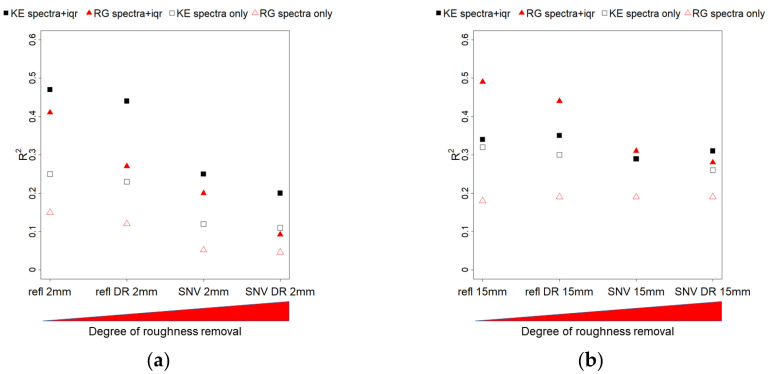
Validation of the PLSR models (using R^2^) for the different data transformations, ordered by their degree of roughness removal, from left (none) to rigth (max). Black symbols depict KE models R^2^, while red symbols represent RG models R^2^. Solid symbols represent models based on spectral data + iqr, void symbols represent models based on spectra only. Panel (**a**) shows result for the 2 mm scale, panel (**b**) results for the 15 mm scale.

**Figure 15:**
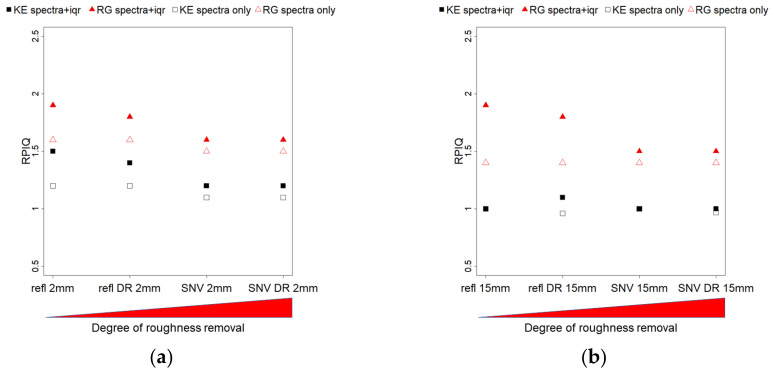
Validation of the PLSR models (using RPIQ) for the different data transformations, ordered by their degree of roughness removal, from left (none) to rigth (max). Black symbols depict KE models R^2^, while red symbols represent RG models R^2^. Solid symbols represent models based on spectral data + iqr, void symbols represent models based on spectra only. Panel (**a**) shows result for the 2 mm scale, panel (**b**) results for the 15 mm scale.

Results from the validation phase of the PLSR models are shown in Figure 14 (R^2^) and Figure 15 (RPIQ). At the 2 mm resolution (Figure 14a and Figure 15a), models for KE performed better when using the combination of pure reflectance + iqr data, while the DR pre-treatment did not affect performance (R^2^ refl = 0.47, refl DR = 0.44). SNV transformation substantially decreased performance both for the base and DR sub-datasets (R^2^ SNV = 0.25, SNV DR = 0.2), on par with the reflectance models based on spectra only (R^2^ refl = 0.25, refl DR = 0.23). Regarding roughness, model performance decreased with the different techniques used to account for roughness effects: in order, from the base reflectance dataset (R^2^ = 0.41) to its DR sub-dataset, to SNV, and finally SNV DR (R^2^ = 0.09). Performance using spectra only (no iqr) were low in all cases. In general, at the fine scale, the datasets with DR pre-treatment were the ones where the difference between KE and roughness model performances was the highest, both with reflectance and SNV. The roughness removal pre-processing methods were lowering the performance of the roughness models, as expected, but SNV negatively affected KE models too. Since SNV did not affect model performance with hyperspectral data, it is possible that the loss of albedo information from this data transformation technique may be necessary for multispectral modelling of KE. For our experiments, reflectance + iqr based models with DR pre-treatment seemed to be the data processing protocol with the best combination of high predictive performance and high discrimination between KE and roughness.

At the 15 mm resolution (Figure 14b and Figure 15b), KE model performance was on average lower than with the 2 mm resolution with reflectance-based data (R^2^ refl = 0.34, refl DR = 0.35) and slightly better with SNV (R^2^ SNV = 0.29, SNV DR = 0.31). Roughness modelling performance showed the same decreasing power with the data pre-treatment techniques, as seen with the 2 mm resolution, from R^2^ = 0.49 to R^2^ = 0.28. No sensible difference was brought by the DR pre-treatment. Regarding the models based on spectra only, KE models resulted in equivalent performance as with the iqr dataset, while roughness models showed generally poor predictions. In general, at the coarser scale, all models for KE performed equally but showed a very low RPIQ (~1), probably due to the limited number of samples and variability in the calibration dataset. Roughness models, on the other hand, provided acceptable predictions. As a result, the best data processing protocol proposed for the 2 mm resolution dataset could not be confirmed when using the 15 mm resolution dataset.

Overall, SNV + iqr did not offer better model performances than using only base reflectance indexes, especially at the finer scale where richer calibration data where available. Given the good modelling performance with the SNV of hyperspectral data, it is possible that the available multispectral resolution was not suitable for this kind of data transformation for KE predictions. The albedo information contained in pure reflectance data may be critical for KE prediction with multispectral data. Other studies that attempted modelling crust development [17,18] obtained good results (0.86 < R^2^ < 0.94) using specific SWIR hyperspectral bands to predict crusted soil infiltration rates, but did not perform a validation of their models. To our knowledge, no other studies attempted modelling soil crusting with vis-NIR multispectral data.

### 3.5. Multispectral Variable Importance

At the 2 mm spatial scale VIP scores showed some interesting differences in variable selection between KE and RG models (Table 1). When using reflectance data, KE models used a combination of base spectral bands (blue, green, NIR) and iqr indexes while roughness models were mainly dependent on iqr indexes (VIP > 1 for red, rededge and NIR). This supports the hypothesis that emerged from the correlation analysis that the spectral iqr contains useful information to model roughness. The SNV shifted the weight of KE model variable selection from iqr towards pure spectral bands (especially on the rededge channel, as expected from correlation analysis) but had the same effect on roughness models. Given that model performances were substantially lowered for both KE and roughness when adopting SNV, it is possible that this technique effectively accounted for the effects of roughness, but the available multispectral sensor resolution was not suitable for the modelling of KE. 

At the 15 mm resolution, variables behaviour for both KE and roughness models was similar to that at the finer resolution. In general, blue iqr and green iqr indexes were discarded by all models (their correlation was also weaker relative to the other three bands iqr). a possible reason for this, as postulated by [23], is that, due to Rayleigh scattering effects in the atmosphere, greater directional interference from atmospheric scattering in shorter wavelengths can cause a less coherent directional signal from the soil in these channels.

## 4. Conclusions

We have developed PLSR models based on vis-NIR multispectral imaging data to predict the cumulative kinetic energy received by soil samples, as a proxy for soil crust development. The combination of hyperspectral data, multispectral imaging and high-resolution topography from photogrammetry offered insights to identify a suitable data pre-treatment protocol that allows to isolate the effects of rainfall kinetic energy (and hence crusting) from micro-scale roughness in soil reflectance factors. After eliminating shadows, we assigned the residual light scattering effect related to soil surface irregularities (i.e., roughness) in the multispectral spatial domain by calculating the inter-quantile range iqr of the reflectance values in a kernel. A high resolution DTM provided a map to exclude areas where prominent roughness was present (i.e., DR sub-setting). At the 2 mm image resolution, the iqr of all multispectral bands was significantly related to soil roughness. PLSR models suggested that a model based on reflectance + iqr data with DR pre-treatment provides the best combination of high predictive performance and high discrimination between kinetic energy and roughness. SNV provided lower performance for kinetic energy prediction with multispectral data, while it did not deteriorate model performance with hyperspectral data. This suggests that hyperspectral data contains information to model kinetic energy (and thus crusting) from small, specific features and this with a high model performance. In contrast, multispectral datasets have to rely more on albedo information, which is removed by SNV. At the 15 mm image resolution, the same conclusions could not be made: models for roughness performed equally or better than those for kinetic energy and as a result, no clear discrimination between them could be made. This issue was most probably related to the limited sample size used and the non-optimal distribution of kinetic energy values for model calibration and our results are therefore not conclusive. A VIP analysis showed that the variable importance was significantly different between kinetic energy and roughness models based on reflectance + iqr sub-setting, supporting our conclusion on the preferential discrimination technique: while kinetic energy models were also relying on base reflectance information, roughness components were almost exclusively related to some specific iqr indexes. 

As a general conclusion, our experiments suggest that it is possible to model the amount of rainfall kinetic energy received by a soil sample, as a proxy for crust development, from vis-NIR based multispectral imaging. However, there is a caveat: roughness effects on spectral features should be eliminated before modelling. Among the tested methods for roughness effect removal, shadow elimination was found to perform consistently at both 2 mm and 15 mm image resolution; DR sub-setting and iqr calculation can improve spectral discrimination of kinetic energy effects from roughness effects, but a fine image resolution is required (good at 2 mm, insufficient at 15 mm). Although these findings are limited to a single soil type, the methods and analysis proposed in this study provide a first workflow to interpret data from multispectral imaging for the mapping of soil crusting.

## Figures and Tables

**Table 1:** VIP scores from the multispectral data PLSR models of kinetic energy (KE) and roughness (RG) at the two different resolutions. VIP scores higher than 1 are marked with grey colour cells.

		Blue	Green	Red	Reded	NIR	Blue iqr	Green iqr	Red iqr	Reded iqr	NIR iqr
KE 2 mm	refl	1.09	1.33	0.989	0.974	1.14	0.48	0.726	0.938	1.04	1.05
	refl DR	1.14	1.35	0.898	0.812	1.01	0.514	0.769	0.998	1.13	1.12
	SNV	0.815	1.24	0.996	1.29	0.998	0.86	0.864	0.864	0.94	1.01
	SNV DR	0.825	1.34	1.13	1.26	1.01	0.816	0.84	0.796	0.867	0.948
RG 2 mm	refl	0.734	0.906	0.731	0.898	0.974	0.591	0.94	1.18	1.34	1.38
	refl DR	0.857	0.97	0.838	0.723	0.927	0.603	0.915	1.18	1.38	1.32
	SNV	0.497	1.01	1.05	1.24	0.71	0.843	0.883	1.08	1.15	1.27
	SNV DR	0.552	0.964	1.55	1.1	0.69	0.821	0.806	0.938	1.05	1.14
KE 15 mm	refl	1.18	1.15	1.09	0.992	1.03	0.778	0.634	0.822	0.945	1.14
	refl DR	1.25	1.2	0.985	0.949	1.13	0.655	0.592	0.822	0.997	1.06
	SNV	0.821	1.66	1.61	0.947	0.865	0.818	0.412	0.495	0.695	0.772
	SNV DR	0.769	1.64	1.66	0.936	0.876	0.768	0.378	0.487	0.782	0.701
RG 15 mm	refl	0.98	0.982	0.906	0.698	1.13	0.591	0.864	1.09	1.15	1.35
	refl DR	1.02	1.08	0.995	0.694	1.28	0.59	0.754	1.03	1.03	1.25
	SNV	0.835	1.22	1.5	0.797	1.38	0.421	0.607	0.771	0.878	0.976
	SNV DR	0.909	1.32	1.49	0.811	1.38	0.41	0.604	0.709	0.814	0.923

**Figure A1:**
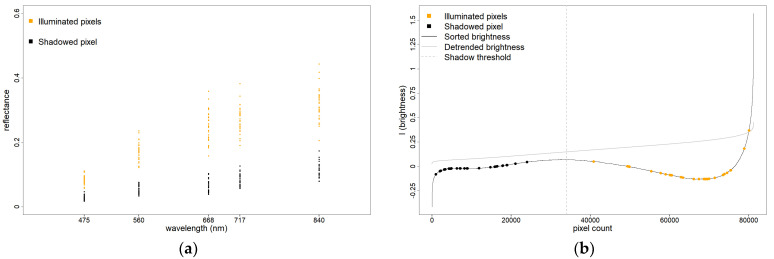
Example of the procedure for shadow threshold individuation on a soil multispectral image: difference in reflectance between illuminated and shadowed calibration pixel (**a**) and the brightness curves after sorting and detrending to make the shadow threshold stand out as a local maximum (**b**).

**Figure A2:**
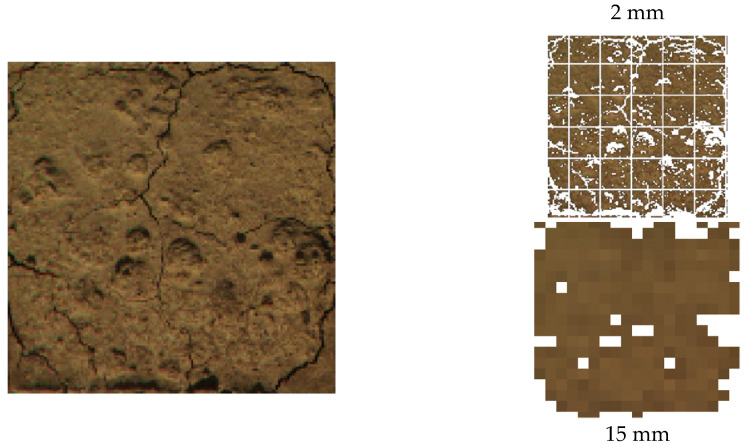
Example of soilbox image (false colour RGB composite) before de-shadowing (**left**), and after de-shadowing at 2 mm resolution (**top right**) and 15 mm resolution (**bottom right**).

**Figure A3:**
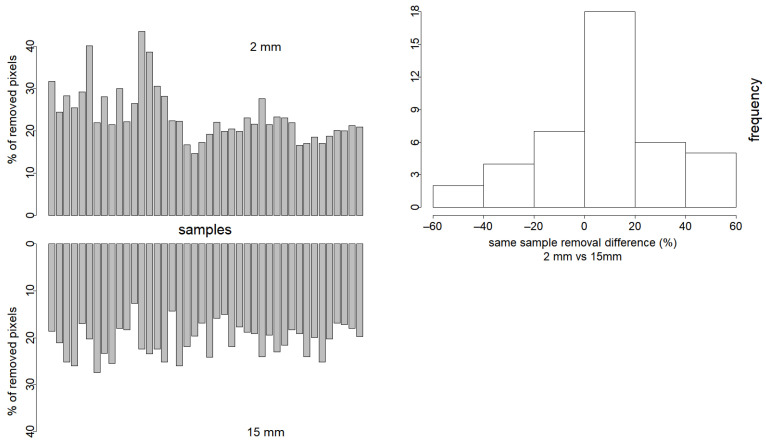
Statistics on the percentage of pixel removal due to shadow across the full dataset: percentage of pixel removal across the samples at 2 mm resolution (**top left**); percentage of pixel removal across the samples at 15 mm resolution (**bottom left**); relative percentage difference in pixel removal between same samples at different resolutions (**right**).

## Data Availability

The data presented in this study are available on request from the corresponding authors.

## Appendix A

### Shadow Removal

A calibration dataset was first created by manually selecting 30 illuminated and 30 shadowed pixels per image on two RGB composite images from two soilboxes (total of 60 and 60 pixels respectively). The difference in reflectance between illuminated and shadowed pixels was evident in all the five spectral bands (Figure A1a), so the brightness index was calculated as Equation (A1):

(A1) $$I=\;\frac{Blue+\;Green+Red+Rededge+NIR}{5}$$

A threshold for automatic unsupervised shadow detection was then individuated by:

(1) sorting all pixel I values pertaining to a soilbox;
(2) applying a detrend function to enhance local peaks by fitting a second order linear model and returning the fitted residuals (Figure A1b);
(3) searching for the first local maximum (vertical grey line Figure A1b).

The threshold was calculated for each single image, and then applied for pixel selection. The quality of the output was visually evaluated in a side-by-side comparison between the original and the de-shadowed image (i.e., Figure A2).

For all soil samples, the percentage amount of pixel removed for the same soil sample between the 2 mm and the 15 mm resolution was compared (Figure A3, left). The relative percentage difference between the removal amount in the two resolutions (Figure A3 right) showed that the majority of samples got almost the same amount of pixels removed (between the −20% and + 20% difference).
